# Rigorous ground plans as terminal taxa: a demonstration in Gastropoda (Mollusca), focusing on Caenogastropoda

**DOI:** 10.7717/peerj.21150

**Published:** 2026-08-24

**Authors:** Luiz Ricardo L. Simone

**Affiliations:** Malacology, Universidade de São Paulo, São Paulo, Brazil

**Keywords:** Phylogeny, Cladistics, Methodology, Taxonomy, Systematics, Malacology, Morphological phylogeny, Morphological data

## Abstract

Despite being widely discussed, the use of ground plans (or ground patterns) as terminals in phylogenetic analyses often faces bias during peer review, as evidenced by criticisms of phylogenetic studies. A ground plan can be defined as the sum of all character states associated with an internal node, representing the synapomorphies and plesiomorphies of a cladogram branch. It is, in fact, a critical outcome of cladistic analysis. Ground plans allow us to theorize about the ancestral species from which a branch evolved and can be effectively employed in subsequent phylogenetic analyses, offering advantages over using sets of actual species. This paper provides a practical example using samples of Gastropoda representatives (Mollusca). The study revisits a large dataset from a cladistic analysis previously published in 2011, which was based on actual species representing the main branches of Mollusca. Here, the main branches of the original cladogram are replaced with their respective ground plans as terminals. The resulting cladogram is presented and compared to the original analysis. All terminals are represented by ground plans, which are discussed in detail. This paper advocates for the broader use of ground plans as terminals in phylogenetic analyses, provided they are derived from a prior, rigorous phylogenetic framework. One major advantage of this approach is the reduced influence of homoplasies, which may be present in actual species but are absent in the ground plan of the taxon it represents, thereby minimizing noise in the final results. The main objective is to mitigate criticisms of using ground plans as terminals in phylogenetic analyses, using a practical and real example as a basis.

## Introduction

The terms “ground plan”, “ground pattern”, and “supraspecific terminal taxa” are commonly used in phylogenetic jargon (*e.g.*, Dupont, 2013; Michalik et al., 2017; Temereva, 2017; Yan et al., 2021). Since Hennig (1965), these terms have referred to a set of fundamental characters that give rise to apomorphic states or represent the common ancestor (*e.g.*, Wheeler, 1998; Smyth, 2018). The concept of a “ground plan” (or “groundplan”) has been interpreted in various ways within biological phylogenetics, ranging from an algorithmic approach (*e.g.*, the groundplan-divergence method—Wagner Jr., 1980; Churchill, Wiley & Hauser, 1984) to the notion of a taxon’s ancestor (*e.g.*, Sene-Silva, 2002; Sinclair & Cumming, 2006; Regier et al., 2015). In this contribution, however, the term “ground plan” adheres to the “exemplar” method outlined by Yeates (1995), which is based on a set of actual representatives of a given higher taxon. This paper also does not deal with the concept of “groundplan-approach”, extracted direct from paleontological data (Kukalová-Peck, 2008; Béthoux, Kristensen & Engel, 2008). Of course, the term “ground plan” is known to carry misconceptions (*e.g.*, suggestions of “primitiveness” or a “fossil-like” condition). However, as will be discussed in due course, replacing this term is difficult in the current context, and addressing these misconceptions is one of the objectives of this paper.

Through the experience of producing and publishing numerous phylogenetic studies on diverse groups of Mollusca (*e.g.*, Simone, 1999; Simone, 2000; Simone, 2001; Simone, 2002; Simone, 2004a; Simone, 2004b; Simone, 2005; Simone, 2006a; Simone, 2006b; Simone, 2007; Simone, 2009; Simone, 2014; Simone, 2017; Simone, 2021; Simone, 2022; Simone, 2023; Simone, 2024b; Ponder et al., 2007; Simone, Mikkelsen & Bieler, 2015; Couto et al., 2016; Dornellas, Couto & Simone, 2020; Pastorino & Simone, 2021) and evaluating feedback and criticisms from reviewers, it has become clear that important insights derived from phylogenetic analyses are often overlooked or disregarded. Each node in a cladogram contains valuable information that could be applied to other phylogenies, rather than relying solely on exemplar species. However, there appears to be a persistent bias against using ground plans (or ground patterns) as terminal taxa to represent supraspecific taxa in phylogenetic analyses, instead of exemplar species. This bias is perplexing, as it is intuitive that the set of synapomorphies and plesiomorphies represented at a node is more effective in depicting a monophyletic branch than a collection of actual species. By incorporating all relevant characters while excluding potential homoplasies within internal branches, this approach reduces noise and enhances the accuracy of the final results.

The term “ground plan” is recognized to carry certain misconceptions, as it may evoke notions of primitiveness or imply a “fossil-like” quality. However, within this context, the term specifically refers to the set of synapomorphies and plesiomorphies associated with a given node. Despite this clarification, the utility of such phylogenetic data requires demonstration through a robust theoretical and practical framework. The aim of this contribution is not to challenge the perspectives of researchers who oppose the use of ground plans but rather to facilitate their broader application in phylogenetic analyses. Ground plans can serve as effective substitutes for sets of actual species or, at the very least, reduce the criticisms often directed at those who employ this approach. This procedure—namely, replacing sets of actual species with their ground plan—is particularly necessary in sequential phylogenetic studies of large taxa, in which multiple papers are produced over time. In such cases, previously studied and well-established monophyletic branches can be represented by their ground plan in subsequent studies and analyses.

A more comprehensive historical perspective and additional bibliographical details on the term “ground plan” can be found in other sources (*e.g.*, Yeates, 1995; Prendini, 2001), making it unnecessary to elaborate on these details here. Moreover, the term is often used in a context that focuses solely on a set of plesiomorphies, which might create confusion with the concept discussed in this article. It is a definition among systematists that the stem species of any existing taxon—whose ground plan represents a hypothesis of the character states it exhibited—is extinct. Beyond to consider nodes as “hypothesis”, this principle is also a cornerstone of Hennigian phylogenetic systematics, as emphasized in Hennig (1966, figs. 4, 6). If this foundational concept is recognized, at least theoretically, it can serve as a basis for broader theoretical investigations.

The use of actual taxa as terminals has been considered to enhance the accuracy of phylogenetic analyses (Wiens, 1998; Bininda-Emonds, Bryant & Russell, 1998; Prendini, 2001) compared to the use of so-called “supraspecific terminal taxa”. However, in cases where supraspecific taxa are employed, the data are often derived by estimating plesiomorphic states from the literature rather than conducting a prior cladistic analysis. This practice has given rise to an alternative methodology, where the most basal actual members of a given taxon are selected to represent it (*e.g.*, Brusatte, 2010; Boyd, 2015). Sometimes the ground plan is represented by a known taxon, like a family or an order, a practice of common use, *e.g.*, in paleontology (*e.g.*, Brusatte, 2010). This approach, however, is not directly applicable to the present study, as it is already present in Simone (2011). In a comprehensive study about supraspecific terminal taxa, it was revealed that that they can lead to biased results (Wiens, 1998), however, if they are based on previous phylogenetic analyses, this method can be considered (Bininda-Emonds, Bryant & Russell, 1998: 129).

One of the most comprehensive phylogenetic analyses based on morphological data was conducted by Simone (2011), involving over 300 taxa and nearly 700 characters, subdivided into approximately 3,000 states. This extensive dataset provides a robust foundation for further experimentation, enabling the testing of alternative approaches and the incorporation of additional taxa. Notably, such expansions have not altered the previous phylogenetic placements (*e.g.*, Simone, 2014; Simone, 2024b; Pastorino & Simone, 2021).

The dataset from Simone (2011) serves as an example of how ground plans can simplify a cladogram, with certain basal branches replaced by their corresponding ground plans. The primary objective of this study was to perform a phylogenetic analysis using ground plans for select mollusk branches, compare the results with those derived entirely from actual species, and illustrate the utility and reliability of applying ground plans obtained in one study to other phylogenetic analyses.

Focusing on ground plans, a more precise definition can be expressed as follows:

\begin{eqnarray*}\sum Si+\sum Pi. \end{eqnarray*}

Where “*Si*” represents the sum of synapomorphies of the branch, and “*Pi*” represents the sum of its plesiomorphies. This definition allows for the theoretical identification of an ancestral species for the given branch, which can then be used as a terminal. While the conventional definition of a ground plan (or ground pattern) typically encompasses the sum of all character states at an internal node, including both apomorphic and plesiomorphic traits, in practice, synapomorphies often receive greater emphasis over symplesiomorphies in certain contexts. The present suggestion clarifies that both types of traits are equally important. Furthermore, the term “synapomorphy” is preferred over “autapomorphy” because the ground plan, as discussed in this paper, mostly refers to a set of taxa, with the shared character state representing a common feature among them. In the present context, the limits of the “ground plan” are therefore purely phylogenetic, corresponding to a taxon, node, or branch in the classical cladistic sense.

The primary objective of this contribution is to evaluate the effectiveness of using theoretical ground plans in phylogenetic analyses. These ground plans, derived from prior phylogenetic studies, replace sets of actual species. The resulting cladograms will be compared and discussed. Phylogenetically-based ground plans can thus be employed in analyses of other taxa as more effective outgroups, potentially minimizing the impact of homoplasies.

## Materials & Methods

**Figure 1:**
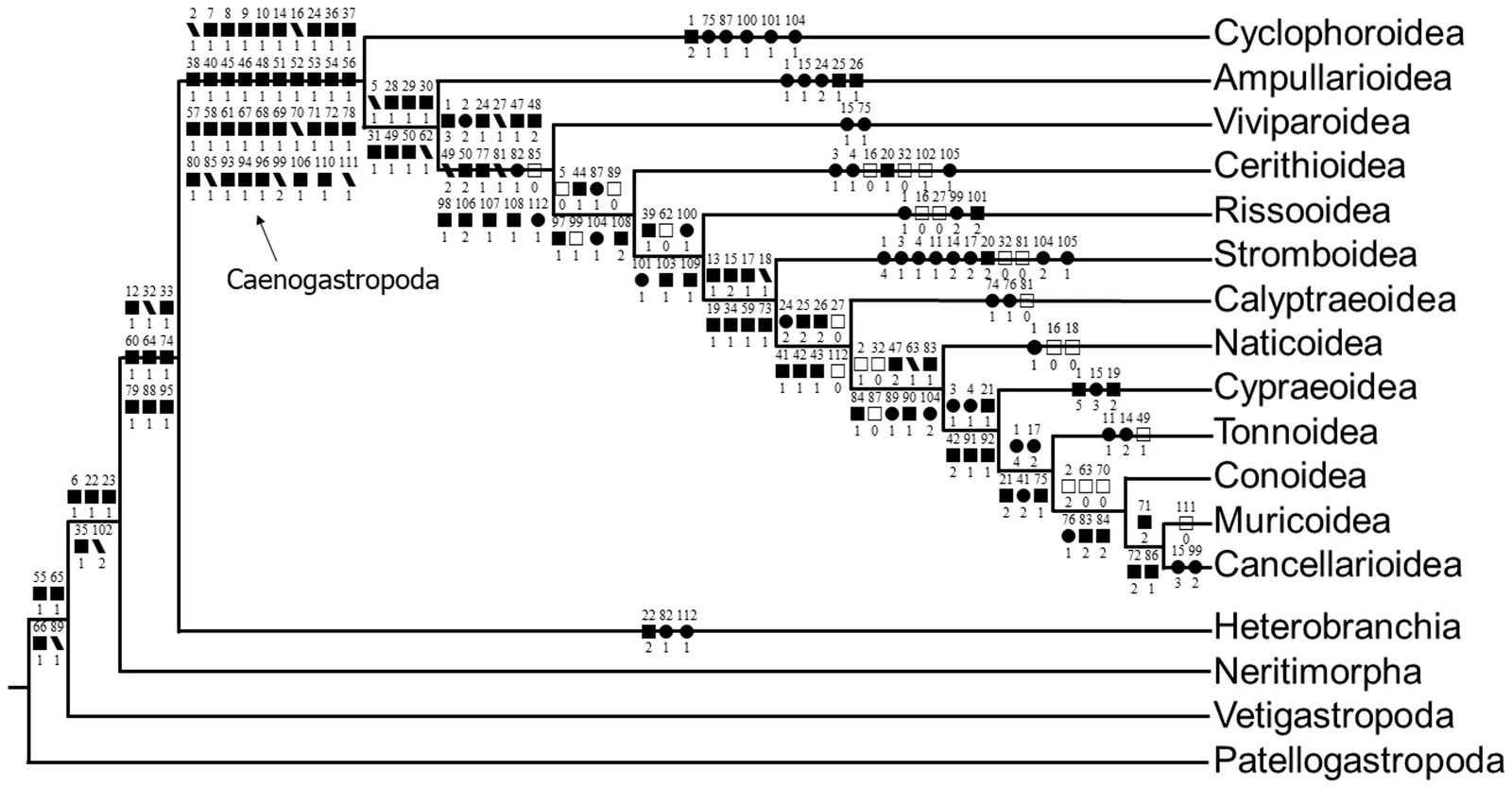
Single most parsimonious cladogram of relationship of Gastropoda (most Caenogastropoda superfamilies) based only on ground plans, synapomorphies of each node indicated. The number on top of each symbol indicates the character; the number under each symbol indicates the state. Outgroup at bottom. Length: 200; CI: 73; RI: 88. Meaning of each symbol: black square, non-homoplactic synapomorphy; black circle, convergence; white square, reversion; inverted dash, synapomorphy that will be reversed.

**Figure 2:**
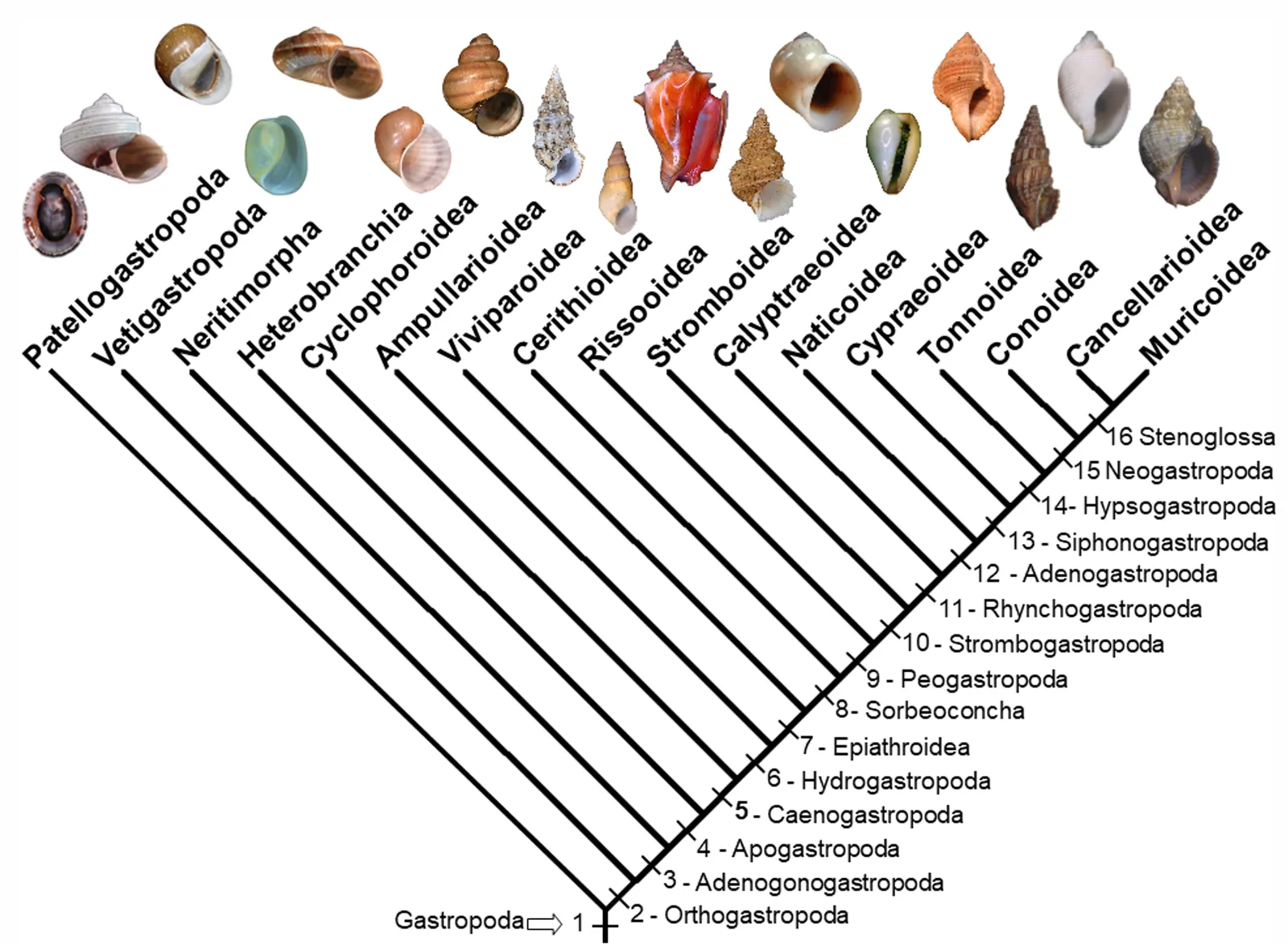
Single most parsimonious cladogram of relationship of Gastropoda main taxa, with named and numbered nodes. Length: 200; CI: 73; RI: 88. In superior region the ground plans (superfamilies and orders) and examples of shells of each terminal (Simone, 2011).

**Figure 3:**
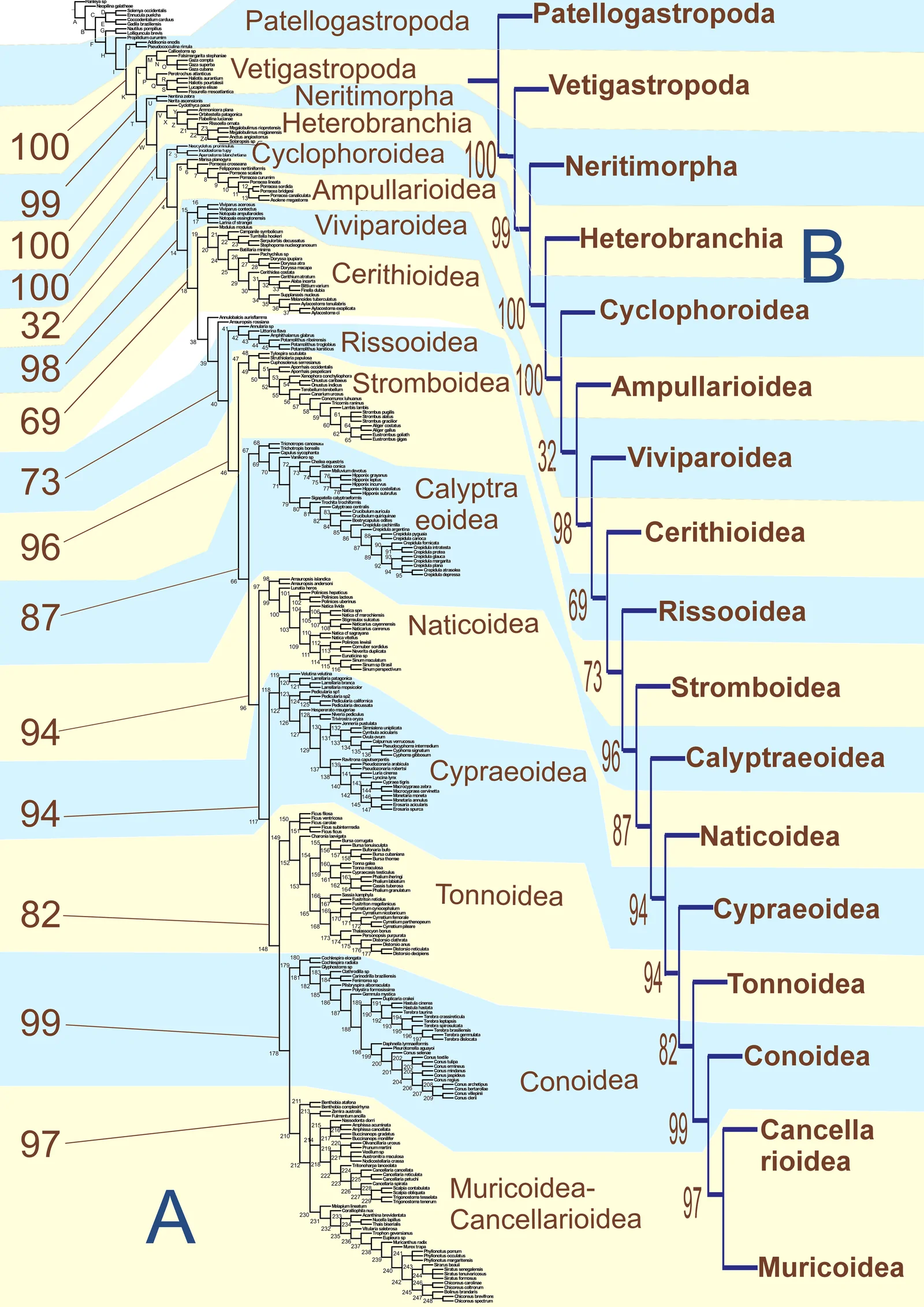
Both studied cladograms with bootstrap values of nodes indicated. (A) Framework of cladogram by Simone (2011, check there for details), with main branches named, only values of basal branches shown. (B) Cladogram.

The phylogenetic framework, including the computer programs and algorithms used, remains consistent with the description provided by Simone (2011: 163–175). This analysis focuses exclusively on Gastropoda, excluding species from other classes. Branches represented by single taxa that form isolated clades are not considered here; this paper focuses only on the main branches. These few branches will be addressed once their representation is increased. Referring to the primary cladogram presented by Simone (2011: 213–216, fig. 20), Table 1 lists the main branches included in this study. The columns specify the node numbering from fig. 20, while the synapomorphies used to define each branch are detailed in Appendix 3 by Simone (2011: 309–317), with the relevant page numbers noted in parentheses in the corresponding columns. A summary of the methodology is as follows: the matrix (Table 2) was constructed using Nexus format (Maddison, Swofford & Maddison, 1997), which was subsequently processed with Winclada (Nixon, 1999) and TNT (Goloboff, Farris & Nixon, 2008; Goloboff & Morales, 2023), all in their latest available versions. The algorithms were calibrated to perform exhaustive replications. After obtaining the cladogram, the apomorphies of each node were examined by tracing the character states along the tree. In the few ambiguous cases, optimization decisions were guided by ontogenetic, developmental, or paleontological evidence, resulting in the cladogram with all synapomorphies of each branch shown (Fig. 1). The same cladogram is exposed in a more didactic manner in order to show the taxonomic definition of each node (Fig. 2). For statistical comparisons, the bootstrap algorithm was employed. The analyses were performed in TNT (Goloboff, Farris & Nixon, 2008; Goloboff & Morales, 2023) using standard procedures and 1,000 replicates. Both matrices were analyzed independently. The matrix of Simone (2011), comprising ∼700 characters and 305 terminals, resulted in bootstrap values for all branches, as previously published in Simone (2011). However, only the branches relevant to the present study are reproduced here (Fig. 3A). The matrix assembled for the present study was analyzed separately, and the resulting cladogram is shown in Fig. 3B.

**Table 1:** Set of taxa considered in the present paper and their data in Simone (2011).

Main collective taxon (alphabetic order)	Branch at Simone’s (2011: fig. 20) cladogram	Set of synapomorphies in the branch indicated in Simone’s (2011) appendix 3, page (item)
Ampullarioidea	5	310 (5)
Calyptraeoidea	67	312 (67)
Cancellarioidea	222	316 (222)
Cerithioidea	19	310 (19)
Conoidea	179	315 (179)
Cyclophoroidea	2	310 (2)
Cypraeoidea	118	313 (118)
Heterobranchia	V	309 (V)
Muricoidea	210	316 (210)
Naticoidea	97	313 (97)
Neritimorpha	U	309 (U)
Patellogastropoda (outgroup)	H	309 (*P. curumim*)
Rissooidea	41	311 (41)
Stromboidea	47	311 (47)
Tonnoidea	149	314 (149)
Vetigastropoda	L	309 (L)
Viviparoidea	15	310 (15)

**Table 2:** Matrix of characters. Taxa listed in first column (outgroup in last line) and character number (see Appendix 1) in first line.

Character		1		2		3		4		5		
	12345	67890	12345	67890	12345	67890	12345	67890	12345	67890	12345	6
Cyclophoroidea	21000	11111	01010	10000	011??	?0???	?1101	11101	00001	10100	11111	1
Ampullarioidea	11001	11111	01011	10000	01121	10111	11101	11101	00001	10111	11111	1
Viviparoidea	32001	11111	01011	10000	01110	01111	11101	11101	00001	11222	11111	1
Cerithioidea	32110	11111	01010	00001	01110	01111	10101	11101	00011	11222	11111	1
Rissooidea	12000	11111	01010	00000	01110	00111	11101	11111	00011	11222	11111	1
Stromboidea	42110	11111	11122	12112	01110	01111	10111	11111	00011	11222	11111	1
Cypraeoidea	51110	11111	01113	??120	11122	20111	10111	12111	12111	12222	11111	1
Naticoidea	11000	11111	01112	01010	01122	20111	10111	11111	11111	12222	11111	1
Calyptraeoidea	32000	11111	01112	11110	01122	20111	11111	11111	11111	11222	11111	1
Tonnoidea	41110	11111	11222	12110	21122	20111	10111	12111	22111	12212	11111	1
Conoidea	42110	11111	01112	12110	21122	20111	10111	12111	22111	12222	11111	1
Muricoidea	42110	11111	01112	12110	21122	20111	10111	12111	22111	12222	11111	1
Cancellarioidea	42110	11111	01113	??110	21122	20111	10111	12111	22111	12222	11111	1
Heterobranchia	00000	10000	01000	00000	02100	00???	?1101	00000	00000	00000	00001	0
Neritimorpha	00000	10000	00000	00000	01100	00000	00001	00000	00000	00000	00001	0
Vetigastropoda	00000	00000	00000	00000	00000	00000	00000	00000	00000	00000	00001	0
Patellogastropoda	00000	00000	00000	00000	00000	00000	00000	00000	00000	00000	00000	0

Character	56		7		8		9		10		11	
	7890	12345	67890	12345	67890	12345	67890	12345	67890	12345	67890	12
Cyclophoroidea	1101	10011	11111	11011	00111	00001	01110	00111	10021	12010	10001	10
Ampullarioidea	1101	11011	11111	11010	00111	00001	00110	00111	10020	02000	10001	10
Viviparoidea	1101	11011	11111	11011	01111	11000	00110	00111	10120	02000	21101	11
Cerithioidea	1101	11011	11111	11010	01111	11000	01100	00111	11110	01011	21201	11
Rissooidea	1101	10011	11111	11010	01111	11000	01100	00111	11121	22110	21211	11
Stromboidea	1111	10011	11111	11110	01111	01000	01100	00111	11111	12121	21211	11
Cypraeoidea	1111	10111	11111	11110	01111	11110	00111	11111	11111	12120	21211	10
Naticoidea	1111	10111	11111	11110	01111	11110	00111	00111	11111	12120	21211	10
Calyptraeoidea	1111	10011	11111	11111	11111	01000	01100	00111	11111	12110	21211	10
Tonnoidea	1111	10111	11111	11111	01111	11110	00111	11111	11111	12120	21211	10
Conoidea	1111	10011	11110	11111	11111	11220	00111	11111	11111	12120	21211	10
Muricoidea	1111	10011	11110	22?11	11111	11220	10111	11111	11111	12120	21211	00
Cancellarioidea	1111	10011	1112?	22?11	11111	11220	10111	11111	11121	12120	21211	10
Heterobranchia	0001	00011	10000	00010	00010	01000	00110	00001	00000	02000	00000	01
Neritimorpha	0000	00001	10000	00000	00000	00000	00010	00000	00000	02000	00000	00
Vetigastropoda	0000	00001	10000	00000	00000	00000	00010	00000	00000	00000	00000	00
Patellogastropoda	0000	00000	00000	00000	00000	00000	00100	00000	00000	00000	00000	00

The plesiomorphies at each node were determined by excluding the synapomorphies and comparing them with those of the sister taxa. The list of characters used in this study focuses on those specifically relevant to the ground plans and is detailed in Appendix 1. Each character is accompanied by its consistency index (CI) and retention index (RI), expressed as percentages under the most parsimonious hypothesis. Additionally, taxa possessing apomorphic states are listed in parentheses, sometimes grouped as a collective taxon. All characters were analyzed using non-additive optimization. Where necessary, a brief discussion is provided for each character, often referring to the final cladogram.

The final cladogram (Fig. 1) was manually arranged for aesthetic purposes based on an optimized cladogram generated by computer programs. Ambiguous states were incorporated after a case-by-case analysis, and any ambiguities are discussed alongside the relevant characters. However, only one optimization is shown in Fig. 1. A second figure (Fig. 2) presents the same cladogram but includes numbered nodes and images of examples of each terminal taxon. The character matrix is shown in Table 2, where inapplicable states are indicated by question marks (?).

The results of the present analysis of ground plans are compared to those of Simone (2011), which are based on actual species. Additionally, personal experiences using ground plans instead of actual species are discussed. While much of the foundation of this study is derived from Simone (2011), other works with relevant phylogenetic information are also considered. These include Simone (1999), Simone (2000), Simone (2001), Simone (2002), Simone (2004a), Simone (2004b), Simone (2005), Simone (2006a), Simone (2006b), Simone (2007), Simone (2009), Simone (2014), Simone (2017), Simone (2021), Simone (2022), Simone (2023), Simone (2024b), Ponder et al. (2007), Simone, Mikkelsen & Bieler (2015), Couto et al. (2016), Dornellas, Couto & Simone (2020) and Pastorino & Simone (2021). These works collectively influence the discussion presented here.

## Results

Focusing on the nodes that form the primary branches of Gastropoda, the more detailed group Caenogastropoda was further subdivided into superfamilies, following the classification system proposed by Simone (2011) (Fig. 3A). Other gastropod groups were classified at the ordinal level. A data matrix was constructed using the ground plans of these main nodes, analyzing their synapomorphies and corresponding symplesiomorphies, as detailed in Table 2.

The analysis of the dataset, which includes 112 characters and 259 states (Table 2), resulted in a single cladogram (Fig. 1, Table 2) with 17 terminals, generated across both computer programs. The cladogram has a length of 200 steps, with a consistency index (CI) of 73 and a retention index (RI) of 88. Figure 1 illustrates the cladogram, highlighting the synapomorphies at each node and the terminal taxa. While most autapomorphies were excluded, those shown in Fig. 1 represent either homoplasies or unique states.

## Discussion

The cladogram published by Simone (2011) primarily aimed to clarify the main branches of Mollusca, with a particular focus on Caenogastropoda, using representative species. This approach recognized that Caenogastropoda account for roughly half of Mollusca diversity. In contrast, the present study focuses exclusively on Gastropoda, analyzing its main branches, which largely correspond to orders and superfamilies. In this context, these branches were replaced with their ground plans as defined in Simone (2011) (Fig. 3A).

The cladogram obtained (Figs. 1, 2 and 3B) aligns with the one presented by Simone (2011, Fig. 20; Fig. 3A) when taxa below the level of superfamilies are disregarded. As a result, the current terminals are represented solely by ground plans, derived from the synapomorphies and plesiomorphies of the corresponding nodes in the previous analysis.

An analysis of the obtained cladogram highlights the utility of ground plans as a simplification method for constructing frameworks in higher classifications. Ground plans can effectively replace the set of representative taxa from which they are derived. However, it is important to emphasize that ground plans are only applicable when preceded by a comprehensive phylogenetic analysis. Such an analysis is essential for establishing the synapomorphies and plesiomorphies that form the foundation of the theoretical taxon. Even studies that argue against the use of ground plans (*e.g.*, Bininda-Emonds, Bryant & Russell, 1998) note that their use becomes more reliable when they are based on prior phylogenetic analyses.

When a specific branch is used in subsequent studies, such as serving as an outgroup, employing ground plans instead of a set of actual species offers several advantages:

1. **Cleaner and clearer analysis**: Ground plans enable a greater focus on higher taxa, reducing the noise caused by secondary modifications. Homoplasies are addressed separately in studies on secondary taxa.
2. **Minimized impact of homoplasies**: Secondary modifications within a taxon do not affect its foundational traits. Using a single representative species risks reversing the polarization of certain characters due to homoplasies in individual terminals. An example is the loss of determinate growth in some neogastropods, such as in buccinanopsids and melongenids. This represents a reversion (Simone, 2011; Simone, 2021); however, if the taxon Neogastropoda is represented by its ground plan, this reversion does not appear. If an actual buccinanopsid or melongenid species is chosen to represent Neogastropoda, indeterminate growth would incorrectly be interpreted as a neogastropod character.
3. **Versatility as outgroups or ingroups**: Ground plans can effectively serve as outgroups—or even ingroups in some cases—offering the combined benefits outlined above. Since the outgroup analysis is based on a comprehensive study of a large set of species, the ground plan represents the entire branch as a single unit. This approach prevents internal homoplasies within the outgroup from influencing the ingroup analysis, as such homoplasies are addressed in the original study that generated the ground plan. An example of using ground plans as outgroups was successfully demonstrated in previous studies. In the phylogeny of the Cypraeoidea (Simone, 2004b), the ground plan of Stromboidea, published in a separate work (Simone, 2005),was tentatively included as a terminal taxon. The resulting analysis placed the Stromboidea ground plan as the sister group to Cypraeoidea (Simone, 2004b: fig. 530B). Another example can be found in the analysis of architaenioglossans (a paraphyletic taxon) (Simone, 2004a), where the insertion of the ground plan of the Cerithioidea (Simone, 2001)confirmed the paraphyly of this taxon (Simone, 2004a, fig. 353). More recently, Simone (2024b) used the ground plan of Strophocheilidae (obtained by Simone, 2022) as an outgroup in a phylogenetic analysis of a sample of Orthalicoidea (Simone, 2024b: fig. 61).
4. **Testing ingroup monophyly**: The use of ground plans facilitates testing the monophyly of an ingroup. By adding ground plans or outgroups (instead of randomly chosen species) as terminals, the analysis can reveal the ingroup’s monophyly if these ground plans fall outside the ingroup’s cladogram.
5. **Enhanced robustness**: Incorporating ground plans based on previous phylogenetic treatments strengthens the analysis, as they are derived from a large set of species, far exceeding the scope of a few selected actual species. The number of characters per taxon usually is larger.

Another advantage is computational efficiency (*e.g.*, reduced computation time and analytical complexity, despite of secondary importance nowadays). In the present case, the streamlined matrix (112 characters/259 states), compared with the full matrix (nearly 700 characters/3,000 states), is far more straightforward to analyze. However, given the computational capacity of modern computers, differences of only a few microseconds are of secondary importance. This is reflected in the cladogram metrics: the present analysis yields a consistency index of 73, which is higher than that of the cladogram based on actual species (CI = 51; Simone, 2011). This provides additional support for the hypothesis that using ground plans effectively reduces homoplasy (“noise”) caused by secondary modifications in individual species.

A concrete example of the “noise reduction” is the superfamily Calyptraeoidea in the present cladogram, which has as a basal synapomorphy the presence of a hairy periostracum. This character also occurs in some secondary branches of the original analysis, such as, *e.g.*, in certain conids (Conoidea) and in cymatiids (Tonnoidea). However, because Conoidea and Tonnoidea are represented here only by their ground plans, the hairy periostracum is absent from these taxa in the present analysis, as this condition occurs only in their derived branches.

In this scenario, the hairy periostracum is therefore not treated as a homoplastic character, and its indices reach 100. In the former analysis, the same character showed several convergences. Its importance as a synapomorphy of Calyptraeoidea remains unchanged, but its indices were around 60 due to the interference of secondary nodes. This effect is called “noise”, which is an unnecessary increase in analytical complexity when the focus is restricted to basal nodes.

Regarding the disadvantages of using ground plans as terminals, few significant drawbacks can be identified (though see below). The primary potential disadvantage is the need for a preceding phylogenetic analysis. Without such an analysis, using a ground plan would be imprudent and open to criticism.

In theory, it should be possible to analyze families or superfamilies in isolation, with each analyzed individually in successive papers. In the final stage, these analyses could be combined based solely on their ground plans, forming the foundation of the overall analysis. This study demonstrates that the outcome is straightforward and may be identical to the analysis using sets of actual species.

In the present analysis, each node in the cladogram (Figs. 1 and 2) is phylogenetically defined by a set of synapomorphies (Fig. 1). For example, the definition of Caenogastropoda (Fig. 2, node 5) is based on 39 synapomorphies (Fig. 1). Similarly, Hydrogastropoda (Fig. 2, node 6) is defined by eight synapomorphies (Fig. 1), Epiathroidea (Fig. 2, node 7) by 17 synapomorphies, and so on. This analysis relies entirely on ground plans. For more details, see Simone (2011) and Simone (2024a).

It is crucial to emphasize that the proposed ground plan is based on a phylogenetic analysis derived from prior studies of actual species. If a phylogeny of a large, monophyletic taxon exists and its ground plan is known, it would be illogical to disregard this valuable dataset in favor of using a small sample of species to represent the taxon in other phylogenies. While there are arguments in favor of using actual species (*e.g.*, Prendini, 2001: 291–295), it is important to note that the current argument is fundamentally rooted in real species. Therefore, the use of ground plans in this context offers all the advantages outlined above. The most common criticism of using ground plans is the potential loss of information from excluding autapomorphies (Prendini, 2001). However, because the ground plans advocated here are derived from previous phylogenetic analyses, information on autapomorphies is already built into them. It is important to recognize that ground plans are not actual taxa, but abstract constructs derived from taxa, like all branches in a cladogram. This should be kept in mind when applying this methodology, although it does not invalidate its use. On the contrary, ground plans can facilitate theoretical reconstruction of the ancestor of a given taxon.

It is important to note that ground-plan concepts may be applied to approaches beyond morphology, including molecular analyses. However, because likelihood and Bayesian algorithms—commonly used in molecular studies—do not generate cladograms based on synapomorphies in the Hennigian sense, the ground-plan concept must necessarily be readapted, mainly if likelihood and Bayesian algorithms are used in non-molecular databases, or in cases of combined molecular and non-molecular matrixes. These issues, however, are under analysis and lie outside the scope of the present paper.

A concise comparison of the obtained cladogram with phylogenies proposed in other recent studies and databases shows that the general framework is similar (Simone, 2024a; Simone, 2024b).Some illustrative examples are discussed below.

In relation to MolluscaBase (2025), some taxa treated there as monophyletic are recovered here as paraphyletic, such as Architaenioglossa (Fig. 2: Cyclophoroidea to Viviparioidea) and Littorinimorpha (Fig. 2: Rissooidea to Tonnoidea). Conversely, Cerithioidea, which is well resolved in the present analysis (Figs. 1 and 2), is considered *incertae sedis* in MolluscaBase (2025).

In comparison with the phylogenomic analyses of Zapata et al. (2014) and Cunha & Giribet (2019), the present study differs in rejecting the monophyly of Patellogastropoda and Vetigastropoda, which here form a paraphyletic arrangement (Figs. 1 and 2). However, the results are congruent in recovering Neritimorpha as the sister group of Apogastropoda, a clade termed Adenogonogastropoda in Simone (2011) and Angiogastropoda in Cunha & Giribet (2019). Those authors referred to the Patellogastropoda/Vetigastropoda branch as Psilogastropoda.

Regarding Neogastropoda, the present arrangement differs from that of Fedosov et al. (2015) and Fedosov et al. (2024) in placing Conoidea at the base of this taxon (see Simone, 2021). Nevertheless, the overall result is fully congruent with most traditional gastropod phylogenetic studies, such as Haszprunar (1988) and Ponder & Lindberg (1997).

Taking into account that any scientific research should be evaluated using quantitative metrics, the bootstrap algorithm was chosen to further compare the cladograms obtained by the two methodologies: analyses based on actual species and analyses based only on their ground plans. As described in the Materials and Methods section, the bootstrap analyses were performed for both datasets in TNT (Goloboff, Farris & Nixon, 2008; Goloboff & Morales, 2023) using standard parameters.

The results are shown in Fig. 3. In Fig. 3A, the database of Simone (2011), comprising ∼700 characters and 305 taxa, was analyzed; only a simplified representation is presented here, and details should be consulted in Simone (2011). Of particular relevance in Fig. 3A are the branches corresponding to the taxa considered in the present study, which are explicitly labeled. Bootstrap values are indicated at the respective nodes. The cladogram obtained in the present study, based on ground plans, is shown in Fig. 3B. Notably, although the two datasets were analyzed independently, the bootstrap values at analogous nodes are identical (Figs. 3A and 3B).

This statistical result further indicates that, even from a mathematical perspective, replacing a monophyletic set of actual taxa with its corresponding ground plan is a consistent procedure.

## Conclusions

1. Ground plans represent significant outcomes of phylogenetic analysis.
2. Ground plans should be defined as the collective sum of both apomorphies and plesiomorphies observed at each node of a derived cladogram.
3. Ground plans can be effectively used as terminals in subsequent analyses, offering advantages over sets of actual species, such as minimizing the impact of secondary homoplasies.
4. The reliability of using ground plans in other analyses is supported by the fact that analyses based solely on ground plans yield cladograms identical to those based on actual species.
5. Therefore, ground plans are an important outcome of phylogenetic analysis and can be further utilized in subsequent analyses and studies.

## Appendix 1—List and Comments of Characters

**1. Shell**

1. Form: 0 = limpet-like or trochiform; 1 = globose (Ampullarioidea, Rissooidea, Naticoidea); 2 = discoid (Cyclophoroidea); 3 = turriform (Cerithioidea, Viviparoidea, Calyptraeoidea); 4 = fusiform (Stromboidea, Tonnoidea, Conoidea, Muricoidea, Cancellarioidea); 5 = involute (Cypraeoidea) (CI = 62; RI = 57).
2. Spire: 0 = about 2 times aperture length; 1 = low (less than half aperture length) (Cyclophoroidea, Ampullarioidea, Cypraeoidea, Naticoidea, Tonnoidea); 2 = very tall (more than 3 times aperture length) (Viviparoidea, Cerithioidea, Rissooidea, Stromboidea, Calyptraeoidea, Neogastropoda) (CI = 50; RI = 71).
3. Determinate growth (well-formed lip): 0 = absent; 1 = present (Cerithioidea, Stromboidea, Cypraeoidea, Tonnoidea, Muricoidea, Cancellarioidea) (CI = 33; RI = 66).
4. Siphon canal (anterior): 0 = absent; 1 = present (Cerithioidea, Stromboidea, Cypraeoidea, Tonnoidea, Conoidea, Muricoidea, Cancellarioidea) (CI = 33; RI = 66).

The shell characters here considered are based on the ground plan of each superfamily. Within each one, the structure presents additional modifications, sometimes totally disfiguring it as, *e.g.*, in the calyptraeoideans. The high plasticity of the shell may be a reflex of the widespread degree of adaptations of the Gastropoda, reached through their evolution (Ponder et al., 2007: Fig. 13.2). Apparently, the shell can change greatly among the taxa, in contrast to the internal anatomy, which has not been modified to the same degree.

**2. Head-foot**

5. Left inhalant siphon: 0 = absent; 1 = present (Ampullarioidea, Viviparoidea) (CI = 50; RI = 0).
6. Eye: 0 = lacking or with open orifice; 1 = with lens (Neritimorpha, Caenogastropoda, Heterobranchia) (CI = 100; RI = 100).
7. Eyes position: 0 = away of tentacles base; 1 = on the tentacle, slightly over its base (Caenogastropoda) (CI = 100; RI = 100).
8. Ommatophore in tentacle: 0 = absent, 1 = present (Caenogastropoda) (CI = 100; RI = 100).
9. Pedal glands: 0 = exposed inside haemocoel; 1 = immersed in the musculature, not seen in the haemocoel floor (Caenogastropoda) (CI = 100; RI = 100).
10. Aperture of the pedal gland: 0 = a pore in the anterior region of the sole; 1 = as a deep furrow along the anterior foot sole (Caenogastropoda) (Heterobranchia = ?) (CI = 100; RI = 100).
11. Distance between head base and furrow of pedal gland: 0 = short; 1 = long (Stromboidea, Tonnoidea) (CI = 50; RI = 0).
12. Number of columellar muscles: 0 = 2; 1 = 1 (Caenogastropoda, Heterobranchia) (CI = 100; RI = 100).
13. Snout/proboscis retractor muscles: 0 = absent; 1 = present, ventro-lateral (Stromboidea, Cypraeoidea, Calyptraeoidea, Naticoidea, Neogastropoda); 2 = present, also dorsal (Tonnoidea) (CI = 100; RI = 100).
14. Septum separating haemocoel from visceral cavity: 0 = absent, 1 = present (Caenogastropoda), 2 = muscular, diaphragm-like (Stromboidea, Tonnoidea) (CI = 66; RI = 75).

**3. Operculum**

15. Form: 0 = circular; 1 = with a projection in the superior and internal quadrant (Ampullarioidea, Viviparoidea); 2 = ovoid or elliptic (Stromboidea, Calyptraeoidea, Naticoidea, Tonnoidea, Conoidea, Muricoidea); 3 = absent (Cypraeoidea, Cancellarioidea) (CI = 60; RI = 71).
16. Outer surface sculpture: 0 = spiral; 1 = concentric (Cyclophoroidea, Ampullarioidea, Viviparoidea, Stromboidea, Calyptraeoidea, Tonnoidea, Neogastropoda); ? = Cypraeoidea, Cancellarioidea (CI = 25; RI = 50).
17. Position of nucleus: 0 = central or sub-central: 1 = eccentric (Naticoidea, Calyptraeoidea); 2 = terminal (Stromboidea, Tonnoidea, Neogastropoda) ? = Cypraeoidea, Cancellarioidea (CI = 66; RI = 87).
18. Occupies entire shell aperture: 0 = yes; 1 = no (Stromboidea, Cypraeoidea, Calyptraeoidea, Tonnoidea, Conoidea, Muricoidea, Cancellarioidea) (CI = 50; RI = 83).

**4. Pallial organs**

19. Mantle border length: 0 = ends in head region during activity; 1 = extends beyond head during activity (Stromboidea, Naticoidea, Calyptraeoidea, Tonnoidea, Conoidea, Muricoidea, Cancellarioidea); 2 = covering shell (Cypraeoidea) (CI = 100; RI = 100).
20. Appendices on mantle border: 0 = absent or complex series of dissimilar papillae; 1 = series of similar-sized papillae (Cerithioidea); 2 = tentacle anterior to anus (Stromboidea) (CI = 100; RI = 100).
21. Distinct siphon in mantle border: 0 = absent, 1 = present, short (Cypraeoidea); 2 = present, very long (Tonnoidea, Neogastropoda) (CI = 100; RI = 100).
22. Number of gills: 0 = 2 (Vetigastropoda); 1 = 1 (Neritimorpha, Caenogastropoda); 2 = 0 (Heterobranchia) (CI = 100; RI = 100).
23. Number of osphradia: 0 = 2; 1 = 1 (Neritimorpha, Caenogastropoda, Heterobranchia) (CI = 100; RI = 100).
24. Type of osphradium: 0 = a node; 1 = long, ridge-like (Viviparoidea, Cerithioidea, Rissooidea, Stromboidea); 2 = elliptical (Ampullarioidea, Cypraeoidea, Calyptraeoidea, Tonnoidea, Neogastropoda) (CI = 66; RI = 90).
25. Osphradium filaments: 0 = absent; 1 = on a fold (Ampullarioidea); 2 = connected directly to mantle by side of osphradial ganglion (Cypraeoidea, Naticoidea, Calyptraeoidea, Tonnoidea, Neogastropoda) (CI = 100; RI = 100).
26. General form of filaments of elliptical osphradium: 0 = absent or ridge-like; 1 = like a fold (Ampullarioidea); 2 = like an ellipse (Cypraeoidea, Naticoidea, Calyptraeoidea, Tonnoidea, Neogastropoda) (CI = 100; RI = 100).
27. Length of osphradium in relation to gill: 0 = much shorter; 1 = almost the same length (Viviparoidea, Cerithioidea, Stromboidea) (CI = 33; RI = 0).

This character presents 2 equally parsimonious possibilities of optimization: (1) state 1 arising in node 3, with a reversion in Rissooidea and in node 7; or (2) convergence among Viviparoidea, Cerithioidea and Stromboidea. The first hypothesis is shown in the cladogram (Figs. 1–2). With the enlargement of the osphradium area, provided by the filaments, the taxa possessing pectinate osphradia (node 7), probably do not require such a long structure.

28. Gill type: 0 = totally or partially bipectinate; 1 = monopectinate (Caenogastropoda) (**?** in Cyclophoroidea) (CI = 100; RI = 100).
29. Gill length: 0 = about 2/3 of pallial cavity length; 1 = about 9/10 of pallial cavity length (Caenogastropoda) (**?** in Cyclophoroidea, Heterobranchia) (CI = 100; RI = 100).
30. Tip of gill filaments: 0 = central; 1 = towards right (Caenogastropoda) (**?** in Cyclophoroidea) (CI = 100; RI = 100).
31. Posterior end of gill: 0 = far from posterior border of pallial cavity; 1 = close to it, almost touching pericardium (Caenogastropoda) (**?** in Cyclophoroidea) (CI = 100; RI = 100).
32. Hypobranchial gland: 0 = thick, with folds and sub-chambers; 1 = thin, with uniform surface (Cyclophoroidea, Ampullarioidea, Viviparoidea, Rissooidea, Calyptraeoidea, Heterobranchia) (CI = 25; RI = 40).

The hypobranchial gland of the vetigastropods and neritimorphs, generally, has

**5. Circulatory System**

33. Number of auricles: 0 = 2 (Vetigastropoda, Neritimorpha); 1 = 1 (Caenogastropoda, Heterobranchia) (CI = 100; RI = 100).
34. Auricle: 0 = free from pericardium anterior wall; 1 = attached to inner surface of pericardium anterior wall (Stromboidea, Cypraeoidea, Naticoidea, Calyptraeoidea, Tonnoidea, Neogastropoda) (CI = 100; RI = 100).

**6. Excretory System**

35. Number of kidneys: 0 = 2; 1 = 1 (Caenogastropoda, Neritimorpha, Heterobranchia) (CI = 100; RI = 100).
36. Relation between kidney and intestine: 0 = intestine runs touching kidney(s); 1 = intestine surrounded by kidney (Caenogastropoda) (CI = 100; RI = 100).
37. Number of renal lobes: 0 = 1; 1 = 2 (Caenogastropoda); 2 = 2 fused in anterior region (Cypraeoidea, Tonnoidea, Neogastropoda) (CI = 100; RI = 100).

This character only refers to the post-torsional left kidney.

38. Inner design: 0 = solid; 1 = some hollow space (Caenogastropoda) (CI = 100; RI = 100).

The kidney of the monotocardians (Caenogastropoda and Heterobranchia) is homologous to the left kidney of the gastropods that possess two kidneys.

39. Nephridial gland: 0 = absent; 1 = present (Rissooidea, Stromboidea, Cypraeoidea, Naticoidea, Calyptraeoidea, Tonnoidea, Neogastropoda) (CI = 100; RI = 100).
40. Nephrostome: 0 = a papilla preceded by renal tissue; 1 = isolated in central region of the membrane between the renal and pallial cavities, free from renal tissue (Caenogastropoda) (CI = 100; RI = 100).

**7. Digestive system**

**7.1. Foregut**

41. Pleurembolic proboscis: 0 = absent (a snout); 1 = short (length equivalent to snout when protracted) (Cypraeoidea, Naticoidea, Calyptraeoidea); 2 = long (more than half of animal length) (Tonnoidea, Neogastropoda) (CI = 100; RI = 100).
42. Situation of retractor proboscis muscle: 0 = absent; 1 = close to apex (Calyptraeoidea, Naticoidea); 2 = close to middle region (Cypraeoidea, Tonnoidea, Neogastropoda) (CI = 100; RI = 100).

The characters 41 and 42 refer to the foregut, however, their analysis can not be be separated from the analysis of some regions of the head (cephalo-pedal mass).

**7.2. Buccal mass**

43. Oral tube (connects mouth with buccal mass): 0 = very short (almost absent); 1 = long (about half of the length of buccal mass) (Cypraeoidea, Naticoidea, Calyptraeoidea, Tonnoidea, Neogastropoda) (CI = 100; RI = 100).
44. Dorsal chamber of buccal mass: 0 = relatively deep (volume almost equivalent to odontophore), 1 = shallow (inconspicuous) (Cerithioidea, Rissooidea, Stromboidea, Cypraeoidea, Naticoidea, Calyptraeoidea, Tonnoidea, Neogastropoda) (CI = 100; RI = 100).
45. Pair of inner folds in dorsal buccal mass wall: 0 = inconspicuous; 1 = broad and evident (Caenogastropoda) (CI = 100; RI = 100).

**7.3. Odontophore**

46. Mj: 0 = diffuse in anterior wall of the buccal mass; 1 = in a form of 2 well defined bundles (Caenogastropoda) (CI = 100; RI = 100).
47. M2: 0 = absent, 1 = present (Viviparoidea, Cerithioidea, Rissooidea, Stromboidea, Calyptraeoidea); 2 = present, also connected to radular sac (Cypraeoidea, Naticoidea, Tonnoidea, Neogastropoda) (CI = 100; RI = 100).
48. M4: 0 = composed of several muscles; 1 = composed of two pairs of superimposed muscles connected to the cartilages (Cyclophoroidea, Ampullarioidea); 2 = composed of a single pair of muscles connected to the cartilages (Viviparoidea, Cerithioidea, Rissooidea, Stromboidea, Cypraeoidea, Naticoidea, Calyptraeoidea, Tonnoidea, Neogastropoda) (CI = 100; RI = 100).
49. Insertion of m4 in the tissue on the radula preceding its exposed (in use) portion (“to” in Figs.) in buccal cavity: 0 = absent; 1 = *via* m9 (Ampullarioidea, Tonnoidea); 2 = *via* ligaments (Viviparoidea, Cerithioidea, Rissooidea, Stromboidea, Cypraeoidea, Naticoidea, Calyptraeoidea, Neogastropoda) (CI = 66; RI = 80).
50. Dorsal and ventral branches of m4: 0 = free from each other anteriorly; 1 = connected anteriorly by means of a cartilage pair (Ampullarioidea); 2 = connected anteriorly (Viviparoidea, Cerithioidea, Rissooidea, Stromboidea, Cypraeoidea, Naticoidea, Calyptraeoidea, Tonnoidea, Neogastropoda) (CI = 100; RI = 100).
51. Ventral tensor muscle of radula: 0 = thick; 1 = very thin (m11) (Caenogastropoda) (CI = 100; RI = 100).
52. Dorsal tensor muscle of radula: 0 = present; 1 = absent (Caenogastropoda) (CI = 100; RI = 100).
53. M5: 0 = originating in cartilages (mainly the posterior cartilages); 1 = originating in m4 (Caenogastropoda) (CI = 100; RI = 100).
54. Form of m5: 0 = short and thick; 1 = long and thin (Caenogastropoda) (CI = 100; RI = 100).
55. Horizontal muscle (m6) situation: 0 = between cartilages; 1 = ventral to cartilages (Vetigastropoda, Neritimorpha, Heterobranchia, Caenogastropoda) (IC = 100; IR = 100).
56. M6: 0 = running along most medial surface of the cartilages; 1 = running less than 2/3 of this surface (Caenogastropoda) (CI = 100; RI = 100).
57. M6: 0 = narrow and thin; 1 = broad and thick (Caenogastropoda) (CI = 100; RI = 100).
58. M7: 0 = absent; 1 = present (Caenogastropoda) (CI = 100; RI = 100).
59. Insertion of m7: 0 = two bundles; 1 = single bundle (Stromboidea, Cypraeoidea, Naticoidea, Calyptraeoidea, Tonnoidea, Neogastropoda) (CI = 100; RI = 100).
60. Approximator muscle of cartilages: 0 = present; 1 = absent or part of m4 (Caenogastropoda, Heterobranchia) (CI = 100; RI = 100).
61. Insertion of m10 in the ventral surface of the odontophore: 0 = posterior; 1 = anterior (Caenogastropoda) (CI = 100; RI = 100).
62. M12: 0 = absent; 1 = present (Ampullarioidea, Viviparoidea, Cerithioidea) (CI = 50; RI = 50).

Although the m12 of the three superfamilies differ in some details, they are suggestively considered homologous. In this point of view, the m12 appear at node 2 and reversed (disappeared) at node 5 and in cyclophoroideans.

63. M14: 0 = absent; 1 = present (Cypraeoidea, Naticoidea, Tonnoidea) (CI = 50; RI = 50).

The pair **m14** is one of the node eight synapomorphies. However, it appears to be absent in neogastropods (node 11), as a reversion.

64. Number of cartilages: 0 = 4; 1 = 2 (Caenogastropoda, Heterobranchia) (CI = 100; RI = 100).

**7.4. Radula**

65. Mineralization of radular teeth: 0 = present; 1 = absent (Vetigastropoda, Neritimorpha, Heterobranchia, Caenogastropoda) (CI = 100; RI = 100).
66. Radular type: 0 = docoglossate; 1 = flexoglossate (Vetigastropoda, Neritimorpha, Heterobranchia, Caenogastropoda) (CI = 100; RI = 100).
67. Subradular cartilage: 0 = narrow; 1 = broad (Caenogastropoda) (CI = 100; RI = 100).
68. Coiling of radular sac: 0 = absent or with ample loops; 1 = intensely coiled when the radular length is sufficient to that (Caenogastropoda) (CI = 100; RI = 100).
69. Number of lateral teeth (in each side): 0 = several (about 5); 1 = 1 (Caenogastropoda **except** Cancellarioidea); 2 = 0 (Cancellarioidea) (CI = 100; RI = 100).
70. Lateral tooth form: 0 = different from rachidian; 1 = tending towards a similarity to rachidian (Cyclophoroidea, Ampullarioidea, Viviparoidea, Cerithioidea, Rissooidea, Stromboidea, Cypraeoidea, Naticoidea, Calyptraeoidea, Tonnoidea) (CI = 50; RI = 80).
71. Number of marginal teeth (on each side): 0 = several (more than 10); 1 = 2 (Cyclophoroidea, Ampullarioidea, Viviparoidea, Cerithioidea, Rissooidea, Stromboidea, Cypraeoidea, Naticoidea, Calyptraeoidea, Tonnoidea, Conoidea); 2 = 0 (Muricoidea, Cancellarioidea) (CI = 100; RI = 100).
72. Form of the marginal teeth: 0 = similar to neighboring teeth; 1 = tending towards a dissimilarity to the neighboring teeth (Cyclophoroidea, Ampullarioidea, Viviparoidea, Cerithioidea, Rissooidea, Stromboidea, Cypraeoidea, Naticoidea, Calyptraeoidea, Tonnoidea, Conoidea); 2 = absent (Muricoidea, Cancellarioidea) (CI = 100; RI = 100).
73. Apex of marginal teeth: 0 = flattened; 1 = sharply pointed (Stromboidea, Calyptraeoidea, Cypraeoidea, Naticoidea, Tonnoidea, Conoidea), (? = Muricoidea, Cancellarioidea) (CI = 100; RI = 100).

**7.5. Salivary glands**

74. Ducts: 0 = absent; 1 = present (Caenogastropoda, Heterobranchia) (CI = 100; RI = 100).
75. General form: 0 = amorphous mass surrounding esophagus and nerve ring; 1 = 2 separated and well delimitated masses (Cyclophoroidea, Viviparoidea, Calyptraeoidea, Tonnoidea, Neogastropoda) (CI = 25; RI = 50).
76. Ducts running: 0 = through nerve ring; 1 = free from nerve ring (Calyptraeoidea, Neogastropoda) (CI = 50; RI = 66).
77. Aperture in dorsal region of buccal mass: 0 = middle level; 1 = rather anteriorized (Viviparoidea, Cerithioidea, Rissooidea, Stromboidea, Cypraeoidea, Naticoidea, Calyptraeoidea, Tonnoidea, Neogastropoda) (CI = 100; RI = 100).

**7.6. Esophagus**

78. Glandular longitudinal bundle running along inner surface: 0 = present; 1 = absent (Caenogastropoda) (CI = 100; RI = 100).
79. Esophageal origin: 0 = middle region of odontophore; 1 = in posterior region of odontophore (Caenogastropoda, Heterobranchia) (CI = 100; RI = 100).
80. Pair of dorsal longitudinal folds in anterior esophagus: 0 = absent; 1 = present (Caenogastropoda) (CI = 100; RI = 100).
81. Pair of longitudinal folds in middle esophagus: 0 = absent; 1 = present (Viviparoidea, Cerithioidea, Rissooidea, Cypraeoidea, Naticoidea, Tonnoidea, Neogastropoda) (CI = 33; RI = 71).
82. Pair of lateral pouches: 0 = present; 1 = absent (Heterobranchia, Caenogastropoda **except** Cyclophoroidea, Ampullarioidea) (CI = 50; RI = 75).
83. Relationship of esophageal gland with esophagus: 0 = diffuse gland (mucosa); 1 = isolated in a large ventral diverticulum (Cypraeoidea, Naticoidea, Tonnoidea); 2 = connected *via* duct (Neogastropoda) (CI = 50; RI = 60).
84. Esophageal gland constitution: 0 = diffuse gland (mucosa); 1 = with transversal septa (Cypraeoidea, Naticoidea, Tonnoidea); 2 = forming acinar sacs (Neogastropoda) (CI = 100; RI = 100).
85. Pair of broad blood vessels connected to the esophageal pouches: 0 = absent, 1 = present (Cyclophoroidea, Ampullarioidea) (CI = 50; RI = 0).
86. Valve of Leiblein: 0 = absent; 1 = present (Cancellarioidea, Muricoidea) (CI = 100; RI = 100).

**7.7. Stomach**

87. Insertion of esophagus in stomach: 0 = posterior; 1 = middle (Cyclophoroidea, Cerithioidea, Rissooidea, Stromboidea, Calyptraeoidea) (CI = 33; RI = 50).
88. Gastric caecum: 0 = present; 1 = absent (Caenogastropoda, Heterobranchia) (CI = 100; RI = 100).
89. Crystalline style sac: 0 = present; 1 = absent (Cyclophoroidea, Ampullarioidea, Viviparoidea, Cypraeoidea, Naticoidea, Tonnoidea, Neogastropoda, Heterobranchia, Vetigastropoda, Neritimorpha) (CI = 33; RI = 50).
90. Stomach size: 0 = broad and large (occupying almost entire visceral mass space where it is located); 1 = narrow (less than 1/3 of that region) (Cypraeoidea, Naticoidea, Tonnoidea, Neogastropoda) (CI = 100; RI = 100).
91. Section: 0 = flat; 1 = approximately cylindrical, having almost same width of adjacent esophagus and intestine regions (Cypraeoidea, Tonnoidea, Neogastropoda) (CI = 100; RI = 100).
92. General form: 0 = blind sac; 1 = a “U” or “V” (Cypraeoidea, Tonnoidea, Neogastropoda) (CI = 100; RI = 100).

**7.8. Intestine**

93. Intestinal loops: 0 = several; 1 = few (generally a single sigmoid loop) (Caenogastropoda) (CI = 100; RI = 100).
94. Most intestine: 0 = immerse in digestive gland; 1 = free from digestive gland, running through kidney and pallial cavity (Caenogastropoda) (CI = 100; RI = 100).
95. Loop inside haemocoel: 0 = present; 1 = absent (Caenogastropoda, Heterobranchia) (CI = 100; RI = 100).
96. Formation of fecal pellets: 0 = absent; 1 = present (Caenogastropoda) (CI = 100; RI = 100).

**8. Reproductive system**

**8.1. Male**

97. Convoluted seminal vesicle: 0 = absent; 1 = present (Cerithioidea, Rissooidea, Stromboidea, Cypraeoidea, Naticoidea, Calyptraeoidea, Tonnoidea, Conoidea, Neogastropoda) (CI = 100; RI = 100).
98. Prostate: 0 = absent; 1 = present in pallial region (Viviparoidea, Cerithioidea, Rissooidea, Stromboidea, Cypraeoidea, Naticoidea, Calyptraeoidea, Tonnoidea, Neogastropoda) (CI = 100; RI = 100).
99. Pallial vas deferens: 0 = absent; 1 = a furrow (Cerithioidea, Stromboidea, Cypraeoidea, Naticoidea, Calyptraeoidea, Tonnoidea, Muricoidea, Neogastropoda); 2 = closed (tubular) (Cyclophoroidea, Ampullarioidea, Viviparoidea, Rissooidea) (CI = 40; RI = 57).

This character refers only to the pallial vas deferens, *i.e.*, which runs along the pallial cavity. This excludes the vas deferens of the heterobranchs, which is haemocoelic.

100. Exophalic penis situated behind and at right from right cephalic tentacle: 0 = absent; 1 = present (Cyclophoroidea, Rissooidea, Stromboidea, Cypraeoidea, Naticoidea, Calyptraeoidea, Tonnoidea, Neogastropoda) (CI = 50; RI = 83).
101. Exophalic penis duct: 0 = penis lacking; 1 = opened (a furrow) (Cyclophoroidea, Stromboidea, Cypraeoidea, Naticoidea, Calyptraeoidea, Tonnoidea, Neogastropoda); 2 = closed (tubular) (Rissooidea) (CI = 66; RI = 83).

**8.2. Female**

102. Pallial oviduct: 0 = absent; 1 = present, with an open duct (a furrow) (Cerithioidea); 2 = present, with a closed duct (tubular) (Cyclophoroidea, Ampullarioidea, Viviparoidea, Rissooidea, Stromboidea, Cypraeoidea, Naticoidea, Calyptraeoidea, Tonnoidea, Neogastropoda, Neritimorpha, Heterobranchia) (CI = 66; RI = 66).
103. Glands of pallial oviduct: 0 = single; 1 = albumen and capsule gland separated from one another (Rissooidea, Stromboidea, Cypraeoidea, Naticoidea, Calyptraeoidea, Tonnoidea, Neogastropoda) (CI = 100; RI = 100).
104. Bursa copulatrix: 0 = absent; 1 = situated in posterior region of pallial oviduct (Cyclophoroidea, Cerithioidea, Rissooidea, Calyptraeoidea); 2 = situated in anterior region of pallial oviduct, originating from genital pore (Stromboidea, Cypraeoidea, Tonnoidea, Neogastropoda) (CI = 100; RI = 100).
105. Furrow running in right side of head-foot, originating from genital pore: 0 = absent; 1 = present (Cerithioidea, Stromboidea) (CI = 50; RI = 0).

**9. Central nervous system**

106. Position of nerve ring: 0 = surrounding mouth; 1 = in middle level of buccal mass (Cyclophoroidea, Ampullarioidea); 2 = posterior to buccal mass (remaining taxa) (CI = 100; RI = 100).
107. Position of pleural ganglia: 0 = close to pedal ganglia (hypoathroid); 1 = close to cerebral ganglia (epiathroid) (Caenogastropoda **except** Cyclophoroidea, Ampullarioidea) (CI = 100; RI = 100).
108. Connectives between the pair of pleural and pedal ganglia: 0 = very short; 1 = long, but separated from the connectives between cerebral and pedal (Viviparoidea); 2 = long, running parallel and close to cerebro-pedal connective (Cerithioidea, Rissooidea, Stromboidea, Cypraeoidea, Naticoidea, Calyptraeoidea, Tonnoidea, Neogastropoda) (CI = 100; RI = 100).

The condition of the heterobranchs is, supposedly, also epiathroid. However, the

109. Statocysts: 0 = with several statoconia; 1 = with a single statolith (Rissooidea, Stromboidea, Cypraeoidea, Naticoidea, Calyptraeoidea, Tonnoidea, Neogastropoda) (CI = 100; RI = 100).
110. Differentiation of buccal ganglia: 0 = diffuse, only a fusiform thickness of buccal nerves; 1 = well defined ganglia (Caenogastropoda) (CI = 100; RI = 100).
111. Position of pair of buccal ganglia: 0 = close to nerve ring; 1 = close to buccal mass (Caenogastropoda **except** Muricoidea) (CI = 50; RI = 75).
112. Buccal ganglia: 0 = one close to another, near median line; 1 = far from one another, situated in lateral region of buccal mass (Viviparoidea, Cerithioidea, Rissooidea, Stromboidea) (CI = 33; RI = 50).

## Additional Information and Declarations

### Data Availability

The following information was supplied regarding data availability: The raw data is available in the Tables, Figures, and Appendices.
